# Absence of DAB2IP promotes cancer stem cell like signatures and indicates poor survival outcome in colorectal cancer

**DOI:** 10.1038/srep16578

**Published:** 2015-11-13

**Authors:** Jiang Min, Liang Liu, Xiaolan Li, Jianwu Jiang, Jingtao Wang, Bo Zhang, Dengyi Cao, Dongdong Yu, Deding Tao, Junbo Hu, Jianping Gong, Daxing Xie

**Affiliations:** 1 Tongji Cancer Research Institute, Tongji Hospital, Tongji Medical College, Huazhong University of Science and Technology, Wuhan 430030, P.R. of China; 2 Department of Gastrointestinal Surgery, Tongji Hospital, Tongji Medical College, Huazhong University of Science and Technology, Wuhan 430030, P.R. of China; 3 Gastrointestinal Surgery Department, The First Affiliated Hospital of ChongQing Medical University, Chongqing 400016, P.R. of China

## Abstract

Metastasis is a critical factor for the high mortality of colorectal cancer (CRC), but its mechanism is not completely understood. Epithelial-mesenchymal transition (EMT) is thought to play a key role in metastasis and also increases the cancer stem cell (CSC) feature that facilitates metastatic colonization. In this study, we investigated the biological roles of DAB2IP regulating EMT and stem cell–like features in human CRC. We demonstrate that DAB2IP suppresses NF-κB-mediated EMT and CSC features in CRC cells. In DAB2IP knockout mice, we discovered the hyperplasia in colonic epithelium which aberrantly represents the mesenchymal feature and NF-κB pathway activation. In clinic CRC tissue, we also reveal that reduced DAB2IP can enrich the CD133^+^ subpopulation. DAB2IP expression was inversely correlated with tumor differentiation and metastasis, and patients with lower DAB2IP expression had shorter overall survival time. Taken together, our study demonstrates that DAB2IP inhibits NF-κB-inducing EMT and CSC to suppress the CRC progression, and also suggests that DAB2IP is a beneficial prediction factor for CRC patient prognosis.

Colorectal cancer (CRC) is one of the most common cancers worldwide^1^, and the second leading cause of cancer-related death in the USA^2^. In its early stages CRC is curable by radical surgical and adjuvant therapies. Nevertheless, treatment in many patients is often precluded by metastasis, thus, understanding the mechanisms of CRC malignancy and metastasis will help develop better therapeutic strategies to improve the rate of treatment.

Increasing evidence indicates that aberrant activation of epithelial-mesenchymal transition (EMT) triggers malignant tumour progression. EMT allows cancer cells to easily detach from primary sites and then invade into lymphatic or vascular systems through loss of epithelial adhesion characteristics and polarity^3^,^4^. Moreover, EMT also induces cells to possess the “stemness” properties to facilitate metastatic lesion in foreign site^5^. These cells, already found in various cancer types, are considered as cancer stem cells (CSCs)^6^,^7^,^8^,^9^. In CRC, CSCs can be enriched by cell membrane proteins such as CD133, CD44, CD166 and ALDH1^8^,^10^,^11^,^12^,^13^. There are many signal pathways involved in regulating EMT and CSC, such as Wnt, Notch, Hedghod, TGF-beta and NF-κB *et al.*^14^, and many EMT activators, such as twist1, snail and ZEB1/2, can induce CSC features^15^,^16^,^17^,^18^. Defining the critical molecules regulating EMT and CSC will definitely provide valuable strategy for CRC therapy.

DAB2IP is first identified as a DOC-2/DAB2 interactive protein and regulated by androgen in prostatic epithelia during castration-induced degeneration. DAB2IP is, also called ASK1 Interacting Protein (AIP1) involved in endothelial apoptosis, a bona fide tumor suppressor and frequently silenced by epigenetic modification in many human tumors^19^,^20^,^21^,^22^,^23^,^24^. Functionally, DAB2IP is a scaffold protein with an activity of GTPase activating protein (GAP) involved in many signaling pathways associated with tumorigenesis including Ras-Raf, PI3K-Akt and ASK1-JNK^25^,^26^,^27^. In addition, DAB2IP was also found to block EMT leading to metastasis^28^,^29^. However, the role of DAB2IP in regulating CSC features and its clinical implication in colorectal cancer has not been determined.

This study identified the key role of DAB2IP, through regulation of the NF-κB pathway, in blocking EMT and suppressing the activity of CSC in CRC. In addition, the expression of DAB2IP positively correlated with 5-year survival of CRC patients. These, in turn, have suggests that DAB2IP is a potent tumor suppressor in CRC and it perhaps can be used as prognostic maker and further developed into a curative therapy for CRC.

## Results

### Absence of DAB2IP facilitates tumor progress in CRC patients

**Figure 1:**
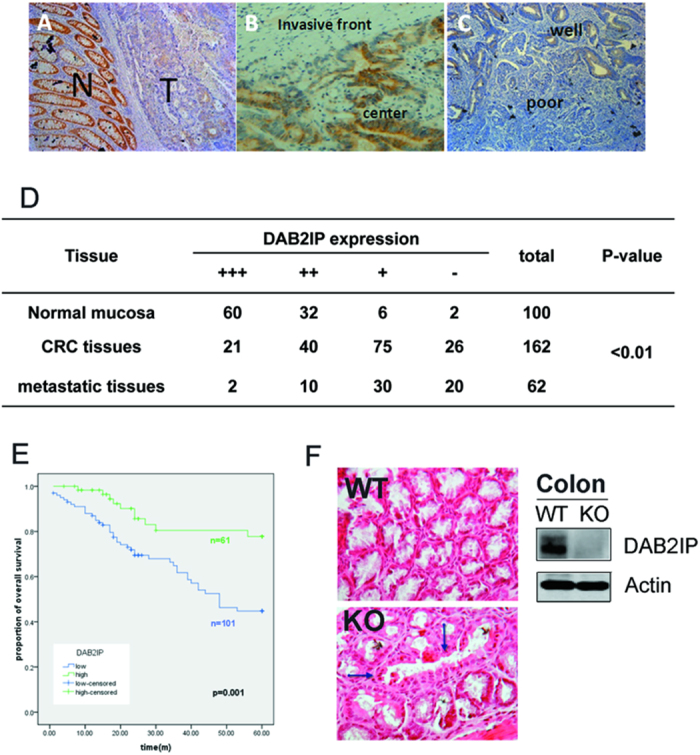
Loss of DAB2IP facilitated the tumor progress in CRC patients. (**A**–**C**) Immunostained comparison of DAB2IP in adjacent normal mucosa and primary site of tumor (**A**); center and invasive front of tumor (**B**); well and poor differentiation (**C**) (magnification: 100x). (**D**) Expressions of DAB2IP in CRC tissues, lymphatic metastatic tissues and adjacent normal mucosa. (**E**) Kaplan-Meier overall survival analysis on colorectal patients classified by total DAB2IP expression in primary site; n = 61 for DAB2IP high group, n = 101 for DAB2IP low group. The ‘censored’ means the researchers does not get the precise survival data because of loss follow-up, accidental death and other reasons. (**F**) Focal aberrant dilated crypt (arrows) in the colonic mucosa from a KO subject (H&E, magnification: 200x).

To assess the significance of DAB2IP in CRC, we first performed an immunohistochemical (IHC) analysis, and found that DAB2IP was distinctly down-regulated in the CRC tissue in comparison with adjacent normal mucosa in the same slices (Fig. 1A), and also observed that expression of DAB2IP in the tumor invasive front was clearly lower than in the center region (Fig. 1B). Therefore, we collected a cohort of CRC tissues (162 cases), adjacent normal tissues (100 cases) and metastatic lesions (62 cases, 57 from lymphatic lesions and 5 from liver metastatic lesions) for IHC analysis. Our data revealed that in adjacent normal mucosa only 8 of 100 cases (about 8%) exhibited low staining (+ or −), while 92 high staining (+++ or ++) (Supplementary Figure 1); in CRC tissue 101 of the total 162 cases (about 62.3%) manifested a low signal; and even 50 of 62 (up to 80.6%) metastatic lesions showed an absent DAB2IP expression (Fig. 1D). Further analysis of the DAB2IP and clinic-pathological factors in the 162 CRC tissues indicated that the loss of DAB2IP strongly correlated with metastasis (P = 0.034) and differentiation (P = 0.004) (Fig. 1C; Supplementary Table 2), while no significant correlation was to be found with age and gender (Supplementary Table 2). Kaplan-Meier curves were used to evaluate the prognostic effects of DAB2IP on CRC patients’ survival and comparisons were made for both high and low DAB2IP groups. Patients with low DAB2IP showed significant lower 5-year survival rates than those with high DAB2IP (P = 0.001) (Fig. 1E). To further determine the physiological role of DAB2IP in colon tissue, we used DAB2IP knockout mice^26^. In DAB2IP knockout mice, colonic crypts lost its monolayer epithelial arrangement and showed aberrant dilated ones (Fig. 1F). Together, these data indicate that DAB2IP is an important prognostic factor and its absence enhances CRC progression.

### DAB2IP regulates EMT in colorectal cancer cells *in vitro*

**Figure 2:**
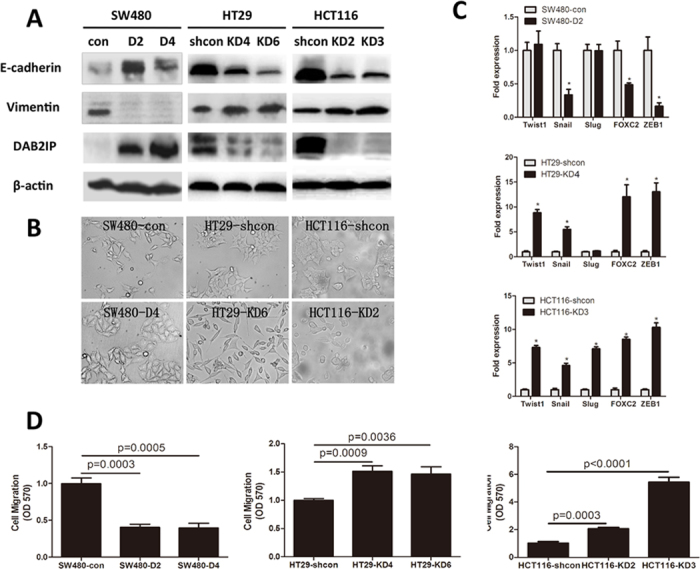
DAB2IP regulates epithelial-mesenchymal transition (EMT) in CRC *in vitro*. (**A**) The stable cell lines were established, SW480 was transfected with either PCI-DAB2IP (D2 and D4) or PCI-neo (control), HT29 and HCT116 with either pGIPZ-shcon(HT29-shcon and HCT116-shcon) or pGIPZ-shDAB2IP(HT29-KD4,KD6 and HCT116-KD2,KD3). E-cadherin, vimentin and DAB2IP were detected, β-actin as loading control. (**B**) The morphology of the sublines was shown by phase-contrast microscopy (magnification: 100x). (**C**) EMT related transcriptional factors were analyzed in SW480, HT19 and HCT116 by quantitative RT-PCR. (**D**) DAB2IP affects the migration *in vitro* by transwell, and quantitative measurement of migratory cell was done.

Next, we profiled DAB2IP, E-cadherin and Vimentin protein expression in three human CRC cell lines. Expressions of DAB2IP and E-cadherin are lower in SW480 than those in HT29 and HCT116 (Fig. 2A). In contrast, Vimentin expression pattern is opposite among these three cell lines. In order to exam whether DAB2IP regulates EMT, two stable DAB2IP-expressing sublines (SW480-D2 and SW480-D4) were generated. The presence of DAB2IP in SW480 increases epithelial phenotypes based on E-cadherin expression (Fig. 2A) and cell morphology (Fig. 2B). In contrast, knocking down the endogenous DAB2IP expression by transfection of GPIZ-shDAB2IP in HT29 and HCT116 promotes EMT (Fig. 2A,B). Transcriptional factors promoting EMT such as Twist1, Snail, Slug, FOXC-2 and ZEB1 were markedly increased after DAB2IP was knocked down, however, over-expression of DAB2IP in SW480 decreased Snail, FOXC-2 and ZEB1 (Fig. 2C). Furthermore, over-expression of DAB2IP can slow down the cell motility of SW480 by transwell migration assay *in vitro*. In contrast, the motility of DAB2IP knockdown HT29 and HCT116 cells appear to increase (Fig. 2D). Taken together, DAB2IP can inhibit EMT and migration in CRC cells *in vitro*.

### DAB2IP modulates the CSC properties in CRC cells

**Figure 3:**
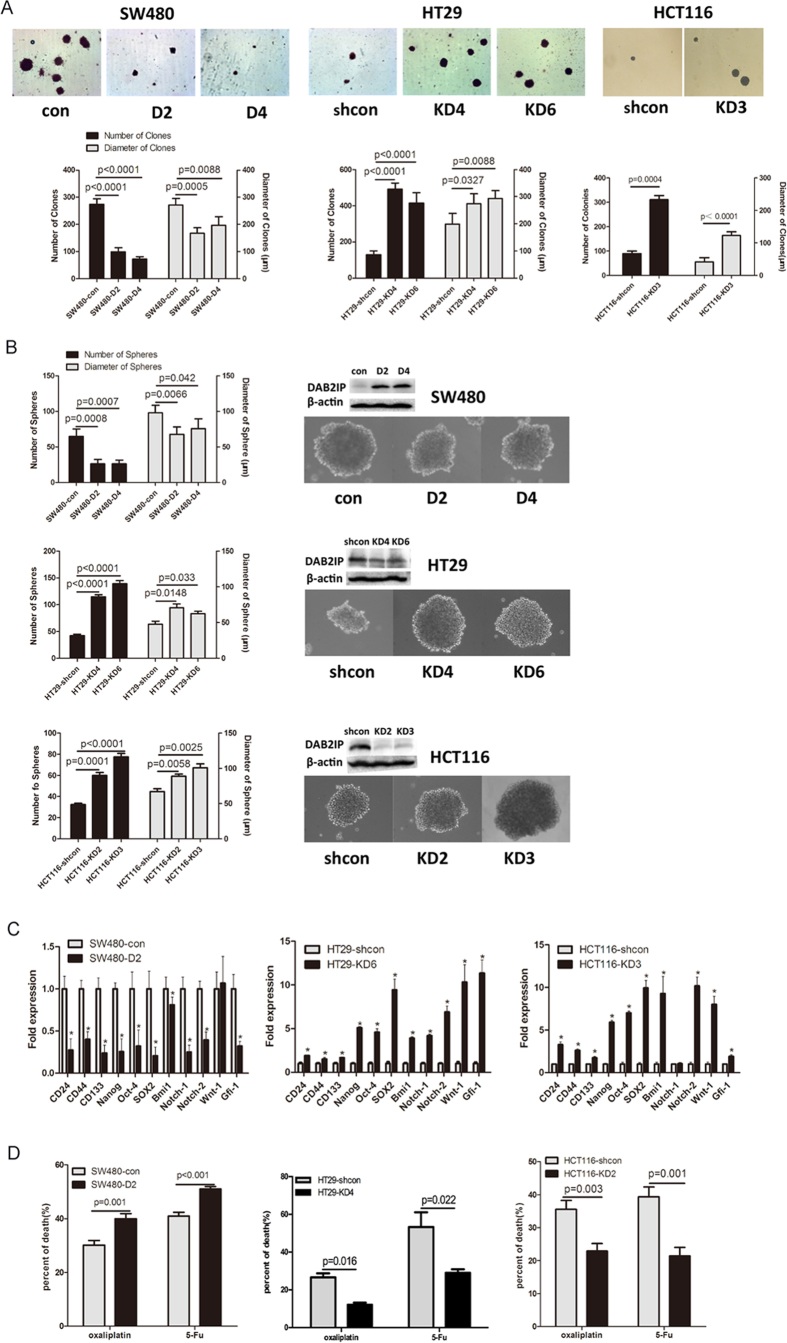
DAB2IP regulates the phenotype of cancer stem cell *in vitro*. (**A**) DAB2IP inhibits clone formation in soft-agar. down: quantitative measurement of number and diameter of clones (diameter> = 50 μm); up: the morphology of clones (magnification: 50x). (**B**) DAB2IP inhibits sphere formation *in vitro*: left: quantitative measurement of number and diameter of spheres (diameter> = 50 μm); right: morphology of spheres (magnification: 100x); western blot showed that DAB2IP expression in sphere cells. (**C**) Surface markers and CSC related transcriptional factors are detected by quantitative RT-PCR. (**D**): SW480 (con and D2),HT29 (shcon and KD6) and HCT116 (shcon and KD2) were treated by oxaliplatin (50 μM) or 5-Fu (200 μg/ml) respectively, after 12 hours, cell death was assayed by PE-Annexin V/7-AAD.

**Figure 4:**
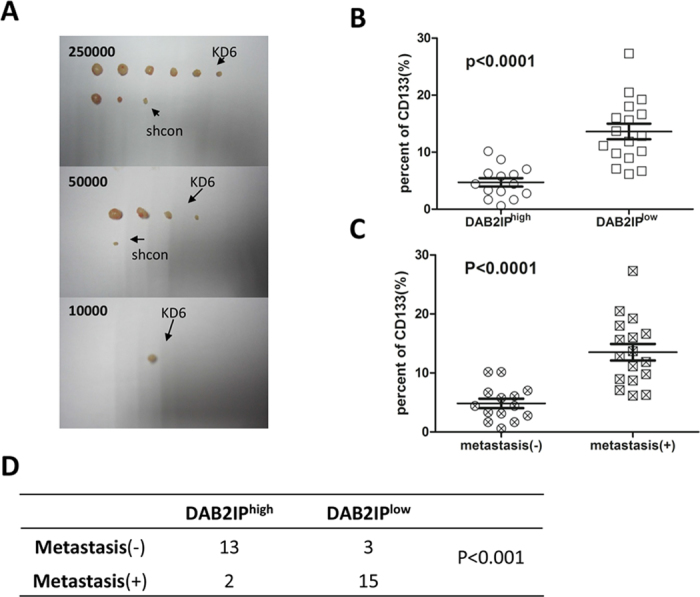
The loss of DAB2IP generated the characteristic of cancer stem cell *in vivo*. (**A**) Count the number of tumor when DAB2IP-knockdown (HT29-KD6) and control cells (HT29-shcon) were transplanted on the different number of 25000, 50000 and 10000. (**B**) Dot plot showed that percent of CD133^+^ cells in high-expression (DAB2IP^high^) and low-expression of DAB2IP (DAB2IP^low^) in colorectal cancer patients. (**C**) Dot plot showed that percent of CD133^+^ cells in no-metastatic (−) and metastatic (+) colorectal cancer patients. (**D**) Four-fold table showed that relationship between DAB2IP expression and metastasis in colorectal cancer patients.

Since DAB2IP is able to prevent EMT, we further determine whether DAB2IP knockdown cells acquire anchorage-independent growth. DAB2IP over-expressing cells (SW480-D2 and SW480-D4) had significantly lower number of clones than the control cells (SW480-con) in soft agar (Fig. 3A). In contrast, DAB2IP knockdown cells (i.e., HT29-KD4, HT29-KD6 and HCT116-KD3) formed both three times more clones than corresponding control cells (Fig. 3A). We further assessed whether DAB2IP could modulate the properties of CSCs based on sphere-forming ability. The presence of DAB2IP reduced the number and diameter of the spheres in SW480 cells. On the other hand, loss of DAB2IP increased the number and diameter of the spheres in HT29 and HCT116 (Fig. 3B). Furthermore, using qRT-PCR to determine the expression level of stem cell markers such as CD24, CD44, CD133, LGR5, ASCL2, DCLK1 and OLFM4, as well as transcriptional factors such as NANOG, Oct4, SOX2, Bmi1, Notch1, Notch2, Wnt1 and Gli-1. Our data indicated that reduced DAB2IP in HT29 and HCT116 cells had elevated expression of these genes (Fig. 3C and Supplementary Figure 2). Knowing that CSCs have an intrinsic resistance to chemotherapy, we compared these cells exposed to oxaliplatin and 5-fluorouracil (5-Fu) at clinically relevant doses. SW480-D2 showed higher sensitivity to both drugs than SW480-con. HT29-KD4 and HCT116-KD2 became more resistant than corresponding control cells (Fig. 3D). Moreover, DAB2IP-knockdown HT29-KD6 facilitated tumorigenicity *in vivo*, compared with control cells (Fig. 4A and Supplementary Table 3). These findings indicate that DAB2IP can suppress CSC-associated features.

### Reduced DAB2IP enriches CD133^+^ cells in CRC patients

It is well known that the subpopulation CD133^+^ CRCs have the same properties as CSC^11^. To analyze whether DAB2IP expression is correlated with the rate of CD133^+^ cells in CRC patients, 31 samples were randomly collected in the same period and later identified as colorectal cancers into different pathological stages. Fresh cancer tissues were separated into single cell suspensions and the percentage of CD133^+^ cells in the viable cancer tissues were directly measured using a Flow Cytometry (Supplementary Table 4). Further analysis showed that the samples of low DAB2IP expression, on the average, had more than a 7% increase in the percentage of CD133^+^ cancer cells than those of high DAB2IP expression (Fig. 4B). Our data also showed that CRC patients with metastasis had about 8% increases of percentage of CD133^+^ relative to those without metastasis (Fig. 4C). In addition, the expression of DAB2IP was found to be negatively correlated with metastasis in 31 cases (Fig. 4D). Together these data suggests that the absence of DAB2IP may enrich the CSC-like cells in CRC patients.

### DAB2IP modulates EMT and stemness through NF-κB pathway

**Figure 5:**
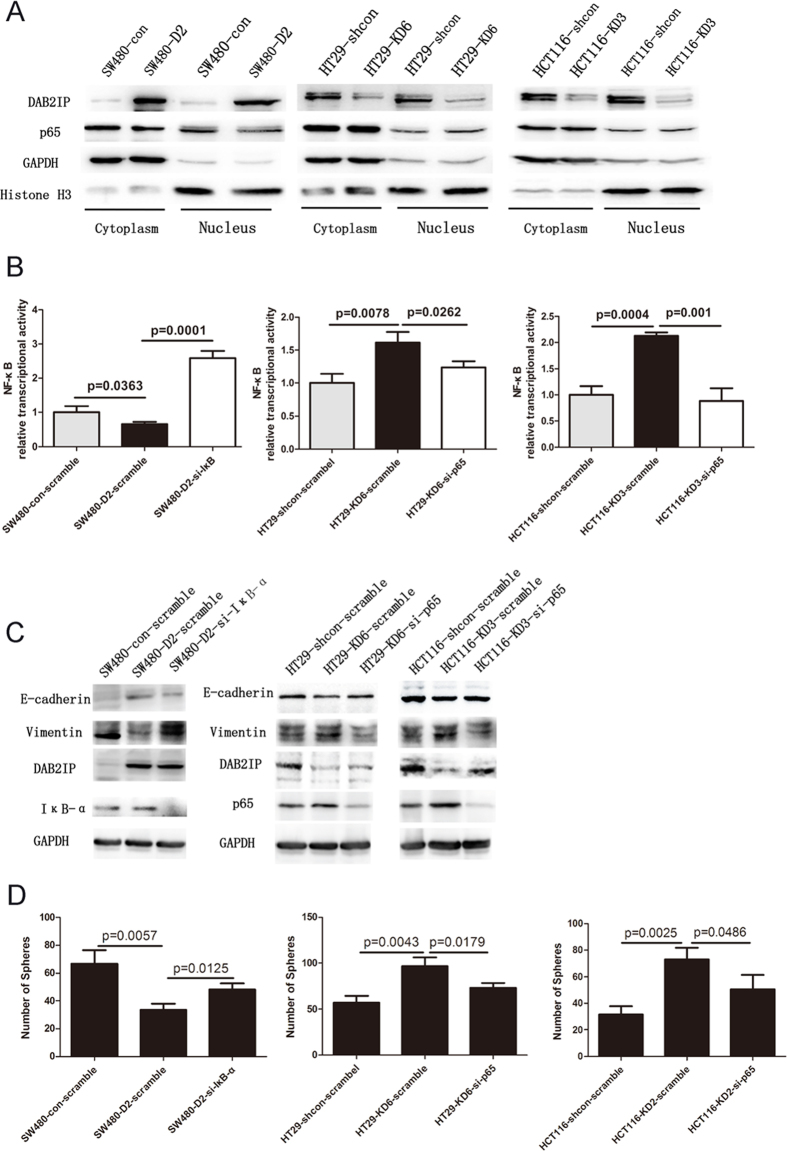
NF-κB pathway mediated DAB2IP-regulated EMT and stemness. (**A**) Nuclear extracts were subjected to western blot analysis and p65 was determined. Histone H3 was used as nuclear marker and GAPDH was used as cytoplasmic marker. (**B**) luciferase reporter assay showed that NF-κB transcriptional activity was reduced in SW480-D2 compared with SW480-con when transiently transfected with scramble siRNA, and was increased in SW48-D2 when transfected with si-IκB compared with SW480-D2-scramble. A strong increase of NF-κB transcriptional activity in HT29-KD6-scramble and HCT116-KD3-scramble was detected, and it was reversed when transfected with si-p65. (**C**) EMT markers were analyzed by western blot in SW480-D2 when treated with scramble or si-IκB, and in HT29-KD6 and HCT116-KD3 treated with scramble or si-p65. (**D**) SW480-D2 was transiently transfected with si-IκB, and quantative analysis of spheres was done compared with SW480-con and SW480-D2 with scramble si-RNA. HT29-KD6 and HCT116-KD2 transfected with si-p65 were analyzed similarly.

To explore the possible mechanism involved in DAB2IP modulation of EMT and stemness, the effects of DAB2IP on NF-κB pathway were examined. In NF-κB pathway, the stimulation from extracellular signals activates receptors and promotes IκB phosphorylation and degradation by a series of signal transmissions, which allows p65 to be released from IκB, and then translocate into the nucleus and initiate the transcription of targeted proteins. First, we purified nucleus proteins in stable DAB2IP over-expressing (SW480) or knocking down (HT29, HCT116) cells and examined p65 protein levels. Our data showed that in comparison with their relevant controls, p65 decreased in SW480-D2 and increased in HT29-KD6 and HCT116-KD3 (Fig. 5A). Next, we detected the activity of DAB2IP on NF-κB pathway by using luciferase reporter assay. Our data indicated that DAB2IP showed an inhibitory effect on NF-κB transcriptional activity (Fig. 5B), thus, DAB2IP could decrease p65 in the nucleus and inhibit the NF-κB pathway. To further confirm whether or not the inhibitory effect of the DAB2IP could be reversed by activating NF-κB pathway, transiently transfected siRNA was used to target IκB (si-IκB-α) in SW480-D2, and scramble siRNA was used to target SW480-con and SW480-D2. The data showed that in SW480-D2-si-IκB-α relative to SW480-D2-scram, that si-IκB-α could reverse the reduced NF-κB activity (Fig. 5B, left). Similarly, when scramble siRNA and NF-κB luciferase plasmid were co-transfected into HT29-shcon and HT29-KD6, and p65 (si-p65) siRNA and NF-κB luciferase plasmid were co-transfected into HT29-KD6, si-p65 reversed the increased NF-κB activity in the HT29-KD6 due to DAB2IP knockdown (Fig. 5B, middle). The same effect was also observed in HCT116 (Fig. 5B, right).

Furthermore, using western blot, the markers of EMT in SW480-con-scramble, SW480-D2-scramble and SW480-D2-si-IκB were detected. Our results showed that NF-κB activation could rescue the E-cadherin increasing and Vimentin decreasing in SW480-D2 (Fig. 5C). Knocking down p65, which appeared to some extent, also recovered the decreased E-cadherin and increased Vimentin in HT29-KD6 and HCT116-KD3 (Fig. 5C). The capacity of sphere formation, on the other hand, was found to be restored when the NF-κB activity in SW480-D2 was activated. Consistently blocking NF-κB in HT29-KD6 and HCT116-KD3 could, however, abolish it (Fig. 5D). Thus, this data clearly indicates the critical importance of the NF-κB pathway for DAB2IP modulation of EMT and stemness in colorectal cancer cells.

### Loss of DAB2IP in CRC promotes cancer metastasis in xenograft model

**Figure 6:**
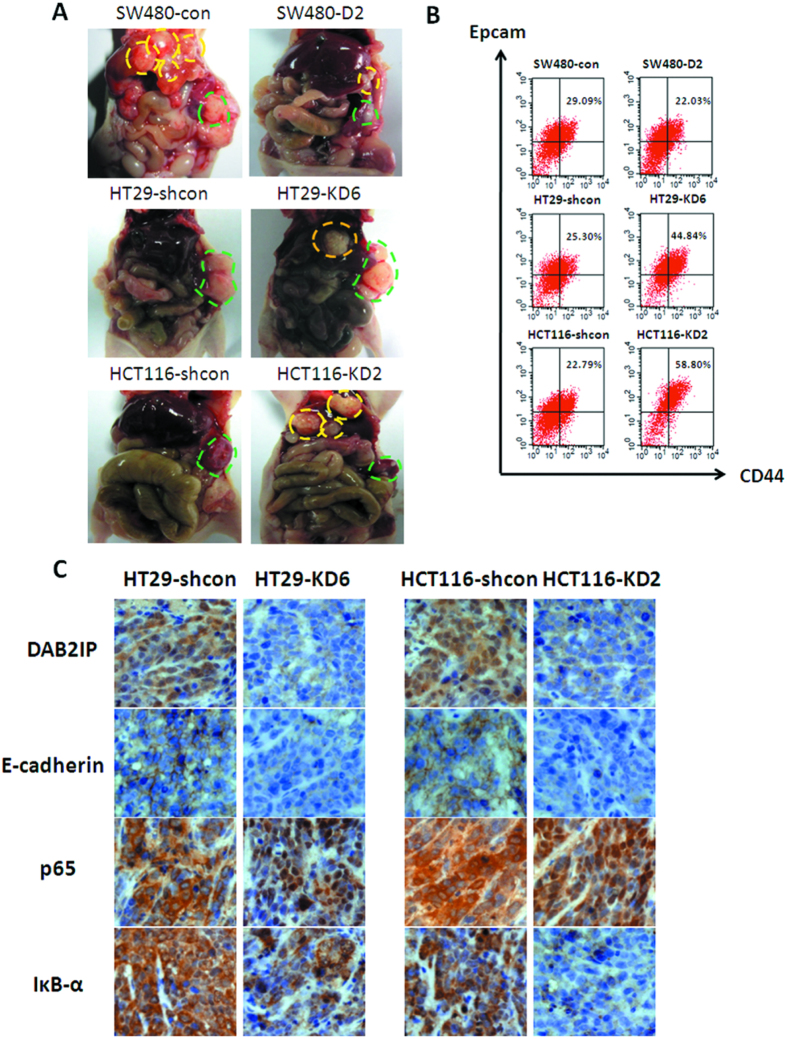
DAB2IP down-regulation promotes metastasis in xenograft model. (**A**) The metastatic lesions were detected live after injection of the clones into the subcapsular region of spleen in nu/nu mice. (2 × 10^6^ sw480, killed after 6 weeks; 10^6^ HT29 and HCT116, killed after 4 weeks) (**B**) Flow cytometry showed the subpopulation Epcam^high^/CD44^+^ in xenograft tumor in spleen. (**C**) Immunostain of paraffin-embedding xenograft tumor from HT29 and HCT116 clones.

A liver metastasis model of colorectal cancer was used to determine the role of DAB2IP in tumor metastasis *in vivo*. Clones were injected into the subscapular regions of mice spleen and allowed to develop for 4 weeks. Then the mice were sacrificed and dissected to investigate any visible metastatic lesions in the liver (Fig. 6A). The data obtained showed that only 2 of the 6 mice bearing SW480-D2 cells underwent liver metastasis, while all 6 of the 6 mice bearing SW480-con cells did. 5 of 6 mice injected into HT29-KD6 had metastasis in the liver, while none of 6 mice showed any metastasis in HT29-shcon. Consistent results were also obtained in HCT116-KD2 (5 in 5) and HCT116-shcon (1 in 6) (Supplementary Table 5). Furthermore, when the tumor tissues were dissociated into single-cell suspensions, the subpopulation of Epcam^high^/CD44^+^ were detected by Flow Cytometry. The results clearly showed that DAB2IP over-expression could decrease the subpopulation of Epcam^high^/CD44^+^ from 29.09% to 22.03% in SW480, and DAB2IP knockdown could increase it from 25.30% to 44.84% in HT29 and from 22.79% to 58.80% in HCT116 (Fig. 6B). In addition, an immunohistochemistry was used to detect the expression of E-cadherin, IκB-α and p65 in those tumors. The absence of DAB2IP promoted the E-cadherin and IκB-α loss, and induced p65 to translocate into the nucleus (Fig. 6C). Together, the animal data forms the conclusion that DAB2IP could impede metastasis *in vivo*.

### Mesenchymal features and activated NF-κB were exhibited in the colonic gland of DAB2IP knockout mice and clinic liver metastatic tissue of patients

**Figure 7:**
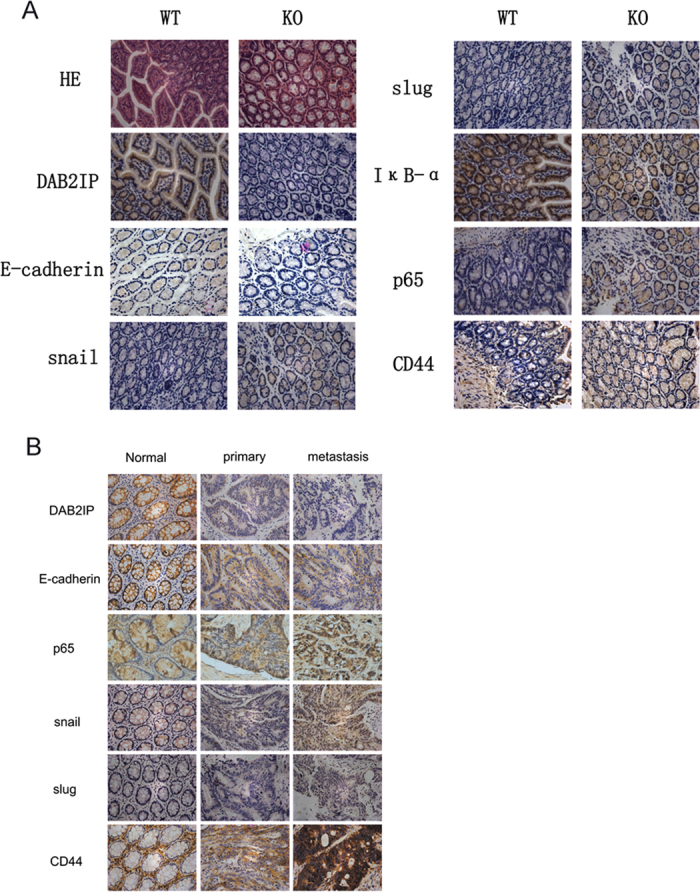
DAB2IP knockout mice exhibited mesenchymal phenotype and loss of DAB2IP facilitated tumor progress in CRC patients. (**A**) HE stain and immunstain analysis of colonic tissue in DAB2IP wild-type and knockout mice. (magnification: 100x). (**B**) Immunostain analysis of adjacent normal mucosa, tumor *in situ* and metastatic lesion in liver from the same patient (magnification: 100x).

To further understand the impact of DAB2IP in colonic tissue, DAB2IP knockout mice (KO; DAB2IP^−/−^) were used. In DAB2IP KO mice, colon tissue showed lower stains of E-cadherin, and transcriptional factors such as Snail and Slug were increased in DAB2IP KO mice (Fig. 7A). IκB was more clearly stained in DAB2IP WT mice colon tissue, however, more CD44 expression and more p65 translocated into the nucleus were detected in DAB2IP KO mice (Fig. 7A). We also investigated expression of these molecules in clinic tissues. DAB2IP was reduced gradually in the adjunct normal tissue, primary tumor and liver metastatic lesion from the same patient (Fig. 7B). Consistently, the decrease of E-cadherin expression was detected in metastatic tissues. However, expression of snail, slug and CD44, as well as the NF-κB signal were activated significantly in metastatic tissues (Fig. 7B). Taken together, the data in DAB2IP KO mice and clinic samples are both consistent with the phonetype in cell lines tests and xenograft model.

## Discussion

In colorectal cancer, genetic events occur in stepwise progression from benign epithelium to CRC, including mutational inactivation of the APC, TP53 and SMAD4 tumor suppressor genes and mutational activation of the KRAS, PIK3CA and BRAF oncogenes^30^. APC, a well known E3 ubiquitin ligase that mediates Wnt/β-catenin degradation^31^,^32^,^33^, truncates frequently in CRC^34^. Previously, our group has demonstrated that DAB2IP could activate GSK3-by forming the complex DAB2IP-PP2A-GSK3-β in prostate cancer^35^. The activated GSK-3β then phosphorylates β-catenin and promotes its binding to APC, which blocks Wnt pathway and EMT via ubiquitination^35^. However, APC truncation can be detected early up to 80% in CRC, which allows β-catenin out of ubiquitination and remains active constitutively^36^. Thus, in SW480 cell which contains 1338-amino-acid truncations on the N-terminal of APC, the Wnt/β-catenin signaling is highly activated. When DAB2IP was over-expressed in SW480 cells, β-catenin transcriptional activity was not changed; however, in HCT116 cells with a wild-type APC, knockdown of DAB2IP leads to a remarkable increase of Wnt activity (Supplementary Figure 3A). These data indicates that in colon cancer cells, the role of DAB2IP in modulating Wnt//β-catenin activity was dependent on the APC status. Nevertheless, regardless of APC status, DAB2IP could still affect the phenotype of EMT and CSC, which takes place through regulating NF-κB pathway (Fig. 5).

NF-κB is a sequence-specific transcription factor that is known to be involved in the inflammatory and innate immune responses^37^,^38^. Recent evidence indicates that NF-κB pathway is also important for tumor progression in CRC^39^,^40^,^41^. Although EMT is usually accompanied by an increase in stem-like properties to facilitate metastatic lesions, the mechanism of NF-κB participating into CSC regulation is not completely disclosed. Activated NF-κB could transcribe an array of secreted proteins such as IL-8, MMPs and VEGF, which involved into EMT^42^. Meanwhile, NF-κB also targets EMT-associated transcriptional factors including TWIST1, SNAIL and Zeb1/2^43^,^44^,^45^, which could regulate some CSC-associated molecules, such as CD24, IL-8, miRNA-200 family *et al.*^15^,^17^,^18^ It was also implied that CD44 is a target of NF-κB-dependent gene expression profile in Hodgkin tumor cells^46^,^47^. In colon and breast cancer, β-catenin could form a complex with NF-κB, resulting in a reduction of NF-κB DNA binding, transcription activity and target gene expression^48^. In this study, we showed the critical role of NF-κB in DAB2IP modulation of EMT and CSC in colon cancer cells (Fig. 5). The mechanism for DAB2IP-regulated NF-κB in CSC needs further study.

As a pioneering work into the clinical application of DAB2IP in CRC, this study indicates the significance of DAB2IP in CRC progression. DAB2IP expression is high in almost all normal mucosa (92/100); while it markedly goes down to about 37.7% (61/162) in CRC *in situ* and about 19.4% (12/62) in metastatic lesions, which suggests that DAB2IP may serve as a basis for CRC occurrence and progression (Fig. 1D). When the clinical characteristics of these patients were assessed with respect to DAB2IP expression, the absence of DAB2IP was found to be positively correlated to poor-differentiation and metastasis which are key factors for CRC prognosis (Supplementary Table 2). In addition, the Kaplan-Meier curves comparing overall survival clearly shows that patients with high DAB2IP had a better than 5-year survival chance (median survival, 41.0 months vs 50.1 months) (Fig. 1E). In metastases-free CRC, the probability of 5-year survival 5 was 100% for DAB2IP-positive and 75% for DAB2IP-negative patients (Supplementary Figure 4). This data clearly indicates that DAB2IP can predict the outcome of CRC patients.

In summary, our study unveils that loss of DAB2IP promoting CRC progression was mediated by activating NF-κB pathway which involves into regulating EMT and CSC signatures. Also, the clinical evidence has lifted the veil on the potential clinical values of DAB2IP in CRC. Since CSCs are resistance to chemotherapy, in order to achieve ultimate cure of cancer, developing stem cell therapy will be a critical strategy. Therefore, DAB2IP not only can be used as a prognostic marker for identifying high-risk patient but also can be developed into tailored therapy.

## Materials and Methods

### Cell lines, plasmid vectors and antibodies

The SW480, HT29, and HCT116 cell lines were purchased from ATCC, and maintained in Dulbecco’s Modified Eagle’s Medium (DMEM; thermo scientific) containing 10% FBS(thermo scientific). PCI-neo and PCI-DAB2IP; pGIPZ-DAB2IP-lentiviral-shRNAmir and pGIPZ-non-silencing-lentiviral-shRNAmir plasmids were gifts from Jer-Tsong Hsieh. Antibodies used in this study were as follows: DAB2IP (gift from Jer-Tsong Hsieh); Vimentin (Cell Signaling); E-cadherin (BD); actin (Santa Cruz); IκB (Santa Cruz); p65 (Cell Signaling); β-catenin (Santa Cruz); PE-CD133 (miltenyibiotec); APC-Epcam (miltenyibiotec) and PE-CD44 (miltenyibiotec).

### RNA isolation and qRT-PCR

RNA was isolated from cell lines using Trizol (invitrogen). For each PCR reaction, 1 μg RNA was reverse transcribed using First Strand cDNA Synthesis Kit (Fermentas). Each cDNA sample was subjected to sequence-specific partial amplification with specific primers (Supplemental Table 1) and the SYBR green PCR Master Mix (Applied Biosystems) on an ABI 7300 plate-reader instrument.

### Cell Migration Assay

5 × 10^4^ cells were plated in the top chamber of a Transwell (24-well insert; pore size = 8 mm; Corning) and incubated with serum-free medium placed in the lower chamber. After incubation for 48 h, cells that did not migrate through the pores were removed by a cotton swab, and cells on the lower surface of the membrane were stained with Cell Stain (Chemicon) and quantified by measuring OD 560 with 96-well plate.

### Clone formation in soft agar

Cells were counted at 10^3^ and plated in EMEM containing 10% FBS and 0.2% agarose (with 0.4% agarose underlay). The number of colonies larger than 100 *μ*m in diameter was determined after 3 weeks.

### Sphere formation

10^3^ Cells were plated with the conditioned medium in 6-well Ultra-Low Attachment plate. The conditioned medium was described^49^. The number of spheres larger than 50 *μ*m in diameter was determined after 3 weeks.

### Luciferase reporter gene assay

Cells seeded in 24-well plates were transfected with 200 ng luciferase reporter plasmids (NF-κB reporter or control) and 1ng of the pRL-SV40 Renilla luciferase plasmid. Total proteins were extracted 24 h after transfection, and the luciferase activity was measured using the Dual-Luciferase Reporter Assay System (Promega).

### Tissue collection, isolation and flow cytometry

All the experimental methods in the current study has been approved by the research committee at Tongji Hospital, Tongji Medical College of Huazhong University of Science and Technology. The study was carried out in accordance with the approved guidelines by the Tongji Hospital Ethics Committee. All patients provided written informed consent. Histological diagnosis was based on microscopic features of carcinoma cells determining the histological type and grade. Cells were labeled with single (anti-CD133-PE) or double (anti-CD44-PE and anti-Epcam-APC) fluorescence for flow cytometry analysis. Isotype-matched IgG was controlled.

### *In Vitro* cell death analysis

2 × 10^6^ Cells were plated in 6-well plate and cultured overnight. Cells were treated with oxaliplatin (50 μM) or 5-Fu (200 μg/ml), after 12 hours, analyzed by PE Annexin V Apoptosis Detection Kit (BD Pharmingen™).

### Animal model

All animal studies were carried out in accordance with the approved guidelines by the Tongji Hospital Ethics Committee. The spleen of nu/nu mice (6–8 weeks of age) were exposed by midventral incision and injected with 10^6^ (HT29 and HCT116) or 2 × 10^6^ cells (SW480) suspended in 50 μl PBS into the subcapsular region of spleen. After 4 weeks, mice were sacrificed and observed.

### Immunohistochemistry

The sections were deparaffinized and rehydrated, and endogenous peroxidase was inhibited with 0.3% H2O2 methanol. For antigen retrieval, slides were boiled in 0.01 M, pH 6.0 sodium citrate buffer for 20 min in a microwave oven. After blocked with the 5% normal goat serum for 40 min, primary anti-DAB2IP polyclonal antibody (1:200, grant from Dr. Jer-Tsong Hsieh) in blocking buffer was applied and the slides were incubated at 4 °C overnight. EnVision™ Detection Systems (Peroxidase/DAB, rabbit/Mouse, DAKO) was applied in the following steps. Stained grading was according to intensity and extent (Supplementary Figure 1).

### Statistical analysis

All error bars in graphical data represent mean ± SD. Student’s two-tailed t test was used for the determination of statistical relevance between groups with P < 0.05 considered as significant.

## Additional Information

**How to cite this article**: Min, J. *et al.* Absence of DAB2IP promotes cancer stem cell like signatures and indicates poor survival outcome in colorectal cancer. *Sci. Rep.* **5**, 16578; doi: 10.1038/srep16578 (2015).

## Supplementary Material

Supplementary Information
