# Supplementary material for: Absence of DAB2IP promotes cancer stem cell like signatures and indicates poor survival outcome in colorectal cancer
Source: Sci Rep. 2015 Nov 13;5:16578. doi: 10.1038/srep16578 (PMC4643237; doi:10.1038/srep16578)
Supplement: Supplementary Information [file srep16578-s1.pdf]

Supplementary information

Absence of DAB2IP promotes cancer stem cell like signatures and indicates poor survival outcome in colorectal cancer

Jiang Min[1,3*], Liang Liu[1,2*], Xiaolan Li[1], Jianwu Jiang[1], Jingtao Wang[1], Bo Zhang[1], Dengyi Cao[1], Dongdong Yu[1], Deding Tao[1], Junbo Hu[1,2], Jianping Gong[1,2#], Daxing Xie[1,2#]

[1]Tongji Cancer Research Institute, [2]Department of Gastrointestinal Surgery, Tongji Hospital, Tongji Medical College in Huazhong University of Science and Technology, Wuhan 430030, P.R. of China.

[3]Gastrointestinal Surgery Department, The First Affiliated Hospital of ChongQing Medical University, Chongqing 400016, P.R. of China

[*] The authors contributed to the paper equally.

[#]**Correspondence:**

Daxing Xie, M.D., Ph.D., 1095 Jiefang Av. Wuhan, Hubei 430030, China; Phone: 86-027-83665275; Fax: 86-27-83662696; E-mail: xdx618@gmail.com or Jianping Gong, M.D., Ph.D., 1095 Jiefang Av. Wuhan, Hubei 430030, China; Phone: 86-027-83665275; Fax: 86-27-83662696; E-mail: jpgong@tjh.tjmu.edu.cn

Supplementary figures

sFig.1

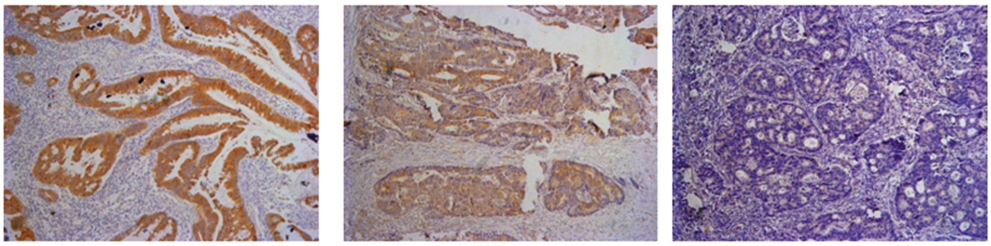

+++ ++ +/-

Supplementary Figure 1: Stained grading of DAB2IP was according to intensity and extent.

sFig. 2

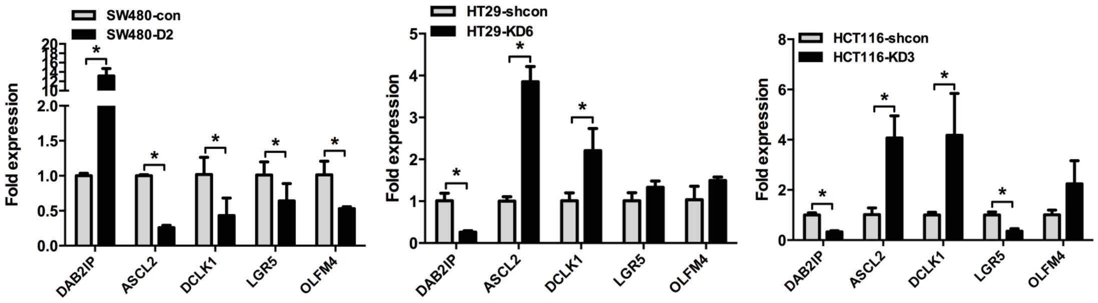

Supplementary Figure 2: Four CSC markers are detected by quantitative RT-PCR.

sFig. 3

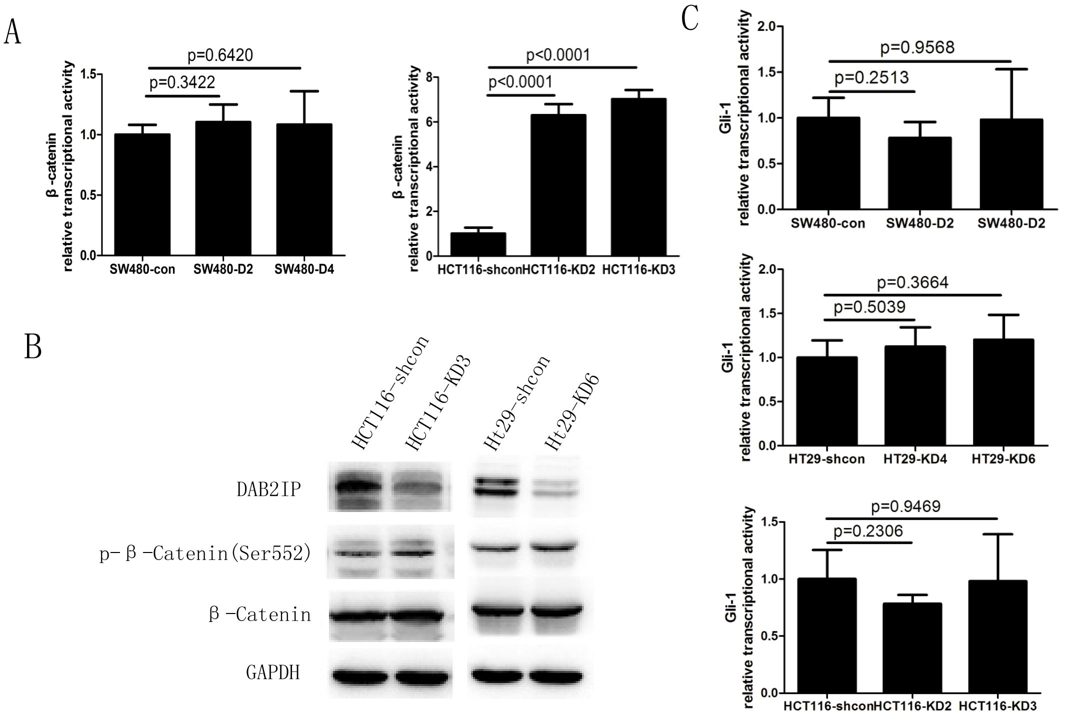

Supplementary Figure 3: (A) β-catenin transcriptional activity was detected by luciferase assay in SW480 and HCT116 cells. (B) p-β-Catenin (Ser552) and total β-Catenin were detected by western blot in HCT116 (shcon and KD3) and HT29 (shcon and KD-6). (C) Gli-1 transcriptional activity was detected by luciferase assay in SW480, HT29 and HCT116.

sFig. 4

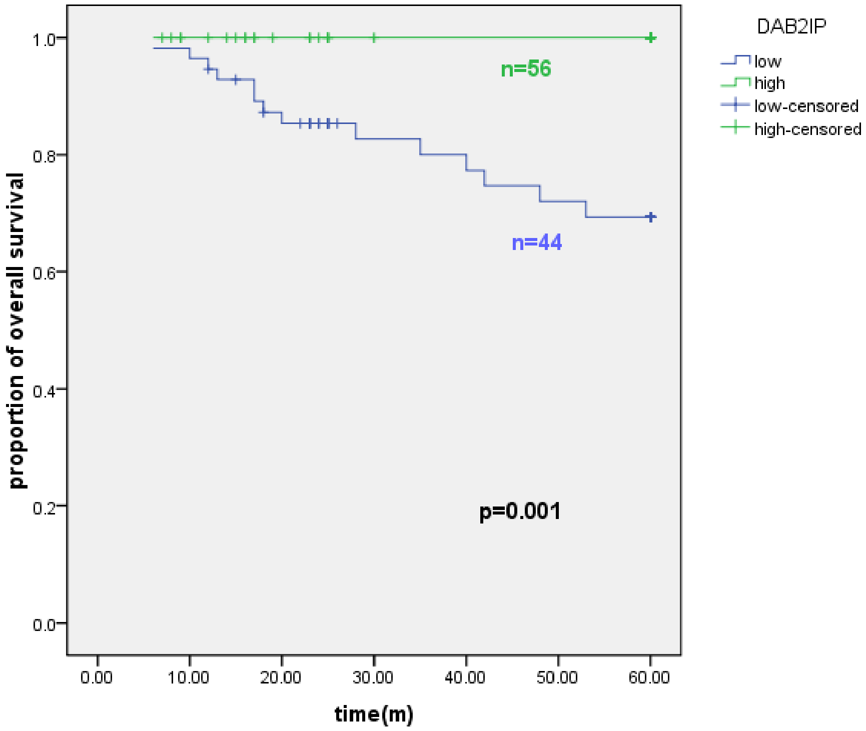

Supplementary Figure 4: Kaplan-Meier analysis on metastases-free colorectal patients classified by total DAB2IP expression in primary site; n = 56 for DAB2IP high group, n = 44 for DAB2IP low group. The ‘censored’ means the researchers does not get the precise survival data because of loss follow-up, accidental death and other reasons.

Supplementary tables

Supplementary Table 1： Primers used in this study

		primers
Twist1	Forward	GGAGTCCGCAGTCTTACGAG
	Reverse	TCTGGAGGACCTGGTAGAGG
Snail	Forward	CCTCCCTGTCAGATGAGGAC
	Reverse	CCAGGCTGAGGTATTCCTTG
Slug	Forward	GGGGAGAAGCCTTTTTCTTG
	Reverse	TCCTCATGTTTGTGCAGGAG
Foxc2	Forward	GCCTAAGGACCTGGTGAAGC
	Reverse	TTGACGAAGCACTCGTTGAG
ZEB1	Forward	TTCAAACCCATAGTGGTTGCT
	Reverse	TGGGAGATACCAAACCAACTG
CD24	Forward	GCCAGTCTCTTCGTGGTCTC
	Reverse	CCTGTTTTTCCTTGCCACAT
CD44	Forward	AGCAACCAAGAGGCAAGAAA
	Reverse	GTGTGGTTGAAATGGTGCTG
CD133	Forward	GCCACCGCTCTAGATACTGC
	Reverse	TGTTGTGATGGGCTTGTCAT
nanog	Forward	CAACCAGACCCAGAACATCC
	Reverse	TTCCAAAGCAGCCTCCAAG
Oct-4	Forward	ACCGAGTGAGAGGCAACC
	Reverse	TGAGAAAGGAGACCCAGCAG
SOX2	Forward	CGAGTGGAAACTTTTGTCGGA
	Reverse	TGTGCAGCGCTCGCAG
Bmi1	Forward	AAATGCTGGAGAACTGGAAAG
	Reverse	CTGTGGATGAGGAGACTGC
notch-1	Forward	ATAGTCTGCCACGCCTCTG
	Reverse	AGTGTGAAGCGGCCAATG
notch-2	Forward	TCCTTCATTTACACAGGGTTCA
	Reverse	GGAGGCGACCGAGAAGAT
Wnt-1	Forward	TAAGCAGGTTCGTGGAGGAG
	Reverse	GGTTTCTGCTACGCTGCTG
Gfi-1	Forward	CCAAGAGTCCCTGGAGCC
	Reverse	AAATCCGAAGGGAAATGAGC
18S	Forward	GGAATTGACGGAAGGGCACCACC
	Reverse	GTGCAGCCCCGGACATCTAAGG

Supplementary Table 2: DAB2IP expression in 162 CRC patients and their relation to clinicopathological factors

feature	Total number	high	low	P-value
Age<br>>=55<br><55	 97<br>65	 35<br>26	 62<br>39	 0.614
Gender<br>Male<br>female	 75<br>87	 34<br>27	 59<br>43	 0.793
Differentiation<br>Well<br>Moderate<br>poor	 47<br>65<br>50	 27<br>20<br>14	 20<br>45<br>36	 <br><u>0.004</u>
Metastasis<br>Y<br>N	 62<br>100	 17<br>44	 45<br>56	 <u>0.034</u>
All cases	162	61	101	

Supplementary Table 3: Subcutaneous injection of tumor cells into nu/nu mice

Cell lines	Number of cells injected		
	10000	50000	250000
HT29-shcon	0/8	1/8	3/8

HT29-KD6	1/8	4/8	6/8

Supplementary Table 4: Case description and patient features

Patient	Age/Sex	Site	Dukes	metastasis	CD133(%)	DAB2IP(IHC)
P1	F	rectum	A	no	0.58	+++
P2	M	rectum	A	no	1.67	+++
P3	F	rectum	A	no	1.67	++
P4	F	transverse	B	no	2.77	+++
P5	M	rectum	A	no	3.11	++
P6	M	rectum	B	no	3.34	++
P7	M	sigmoid	B	no	4.4	++
P8	F	rectum	B	no	4.41	++
P9	M	rectum	B	no	5.75	+++
P10	F	sigmoid	A	no	6.05	++
P11	M	sigmoid	B	no	6.68	+
P12	M	left	A	no	7	++
P13	F	right	B	no	10.16	++
P14	M	rectum	B	no	10.16	+
P15	M	Sigmoid	C	yes	6.16	+
P16	F	transverse	D	yes	6.28	++
P17	F	rectum	C	yes	7.09	+
P18	M	sigmoid	C	yes	8.71	++
P19	M	rectum	D	yes	8.96	+
P20	M	right	C	yes	9.79	+
P21	F	left	C	yes	11.15	+
P22	F	rectum	C	yes	11.87	+
P23	M	rectum	C	yes	12.8	+
P24	M	sigmoid	C	yes	13.7	-
P25	F	rectum	C	yes	15.6	+
P26	M	left-sigmoid	C	yes	16.0	+
P27	M	sigmoid	D	yes	16.63	+
P28	M	transverse	C	yes	18.0	+
P29	F	rectum	C	yes	19.23	+
P30	F	rectum	D	Yes	20.49	+
P31	F	right	D	yes	27.3	+

Supplementary Table 5: metastasis information after DAB2IP overexpression or knock down

Cell lines	Number of mice with liver metastasis
SW480-con	6/6
SW480-D2	2/6
HT29-shcon	0/6
HT29-KD6	5/6
HCT116-shcon	1/6
HCT116-KD2	5/5
